# Understanding Hypoxia-Driven Tumorigenesis: The Interplay of HIF1A, DNA Methylation, and Prolyl Hydroxylases in Head and Neck Squamous Cell Carcinoma

**DOI:** 10.3390/ijms25126495

**Published:** 2024-06-12

**Authors:** Julia Ostapowicz, Kamila Ostrowska, Agnieszka A. Rawłuszko-Wieczorek, Bartosz Wojtera, Sabina Koczot, Wojciech Golusiński, Wiktoria M. Suchorska

**Affiliations:** 1 Department of Electroradiology, Poznan University of Medical Sciences, 61-866 Poznan, Poland; 2 Radiobiology Laboratory, The Greater Poland Cancer Centre, 61-866 Poznan, Poland; 3 Doctoral School, Poznan University of Medical Sciences, 60-812 Poznan, Poland; 4 Department of Head and Neck Surgery, Poznan University of Medical Sciences, The Greater Poland Cancer Centre, 61-866 Poznan, Poland; 5 Department of Histology and Embryology, Poznan University of Medical Sciences, 60-781 Poznan, Poland; arawluszko@ump.edu.pl

**Keywords:** HNSCC, hypoxia, DNA methylation, epigenetics, HIF1A, prolyl hydroxylases

## Abstract

Hypoxia-inducible factor 1-alpha (HIF1A) is a key transcription factor aiding tumor cells’ adaptation to hypoxia, regulated by the prolyl hydroxylase family (EGLN1-3) by directing toward degradation pathways. DNA methylation potentially influences EGLN and HIF1A levels, impacting cellular responses to hypoxia. We examined 96 HNSCC patients and three cell lines, analyzing gene expression of *EGLN1-3*, *HIF1A*, *CA9*, *VEGF*, and *GLUT1* at the mRNA level and EGLN1 protein levels. Methylation levels of *EGLNs* and *HIF1A* were assessed through high-resolution melting analysis. Bioinformatics tools were employed to characterize associations between *EGLN1-3* and *HIF1A* expression and methylation. We found significantly higher mRNA levels of *EGLN3*, *HIF1A*, *GLUT1*, *VEGF*, and *CA9* (*p* = 0.021; *p* < 0.0001; *p* < 0.0001; *p* = 0.004, and *p* < 0.0001, respectively) genes in tumor tissues compared to normal ones and downregulation of the *EGLN1* mRNA level in tumor tissues (*p* = 0.0013). In HNSCC patients with hypermethylation of *HIF1A* in normal tissue, we noted a reduction in *HIF1A* mRNA levels compared to tumor tissue (*p* = 0.04). In conclusion, the differential expression of *EGLN* and *HIF1A* genes in HNSCC tumors compared to normal tissues influences patients’ overall survival, highlighting their role in tumor development. Moreover, DNA methylation could be responsible for *HIF1A* suppression in the normal tissues of HNSCC patients.

## 1. Introduction

Head and neck cancers (HNCs) are the seventh most prevalent malignancies globally, characterized by a high mortality rate with no significant improvement over the past years. Most HNCs are squamous cell carcinoma (HNSCC), which arises from the epithelium of the upper aerodigestive tract [1]. The lack of reliable biomarkers for early detection, treatment monitoring, and follow-up significantly contributes to the poor prognosis, reflected in a mere 40–50% 5-year survival rate. The main risk factors associated with the high morbidity of HNSCC include tobacco smoking and alcohol consumption. Still, in recent years, infection with the Human Papillomavirus (HPV) has emerged as a significant contributor to HNSCC cases, especially oropharyngeal and laryngeal neoplasms [2]. HNSCCs are solid tumors characterized by rapid cell proliferation, associated with insufficient blood supply, leading to hypoxic region development [3]. Within a hypoxic environment, tumor cells exhibit a more aggressive phenotype, resistance to treatment, and induction of metastasis-related processes [4]. Cancer cells adapt to hypoxia by modulating the expression of genes involved in neoangiogenesis (e.g., vascular endothelial growth factor, *VEGF*), glucose uptake (e.g., glucose transporter, *GLUT1*), pH modulation (e.g., carbonic anhydrase IX, *CA9*), and other adaptive mechanisms. Hypoxia-inducible factors (HIFs) play a crucial role in cancer adaptation to lower oxygen concentrations by binding to hypoxia response elements (HREs) in the promoter regions of target genes. HIF comprises two subunits: oxygen-dependent HIF-α and constitutively expressed HIF-β subunit [5]. When exposed to hypoxic conditions, HIF-α and HIF-β form a heterodimer, translocate to the nucleus, and initiate the transcription of target genes [6,7,8].

Under normoxic conditions, the level of HIF-α is regulated by the prolyl hydroxylase enzyme family (PHD) and factor-inhibiting hypoxia-inducible factor (FIH). PHD1, PHD2, and PHD3 (their official names are Egl nine homologs EGLN2, EGLN1, and EGLN3, respectively) hydroxylate conserved prolyl residues at the 402 and 564 positions in the oxygen-dependent degradation domain (ODDD) of HIF-α. The von Hippel–Lindau tumor suppressor protein (pVHL) recognizes hydroxylated HIF-α and is subsequently targeted to the proteasomal degradation pathway [9]. Among the EGLN isoforms, EGLN1 is most active toward HIF1α, whereas EGLN2 and EGLN3 are more efficient in HIF2α hydroxylation. By regulating HIF stability and activity, EGLNs influence key aspects of the tumor microenvironment, including angiogenesis, metabolism, and extracellular matrix remodeling, ultimately contributing to tumor progression and metastasis [10].

Although various studies have demonstrated different expression levels of prolyl hydroxylases and hypoxia-inducible factors in tumors compared to adjacent normal tissues [11,12,13], the mechanism of its regulation remains unclear. DNA methylation, mediated by DNA methyltransferase enzymes, involves adding a methyl group to the carbon-5 position of the cytosine base, primarily within cytosine-guanine dinucleotides (CpG). Most CpG islands are 500 to 1000 bases long and are typically located in the promoter regions of genes. During cancer pathogenesis, there is an alteration in epigenetic mechanisms, leading to widespread changes in normal DNA methylation patterns [14]. Differential DNA methylation patterns occur in various malignancies, including hyper- or hypomethylation [15]. Hypermethylation is common for tumor suppressor genes and genes responsible for DNA repair. Furthermore, decreased DNA methylation is also characteristic of tumor cells, especially in the regulatory regions of the oncogene [15]. We hypothesize that DNA methylation of the CpG island in the promoter region of *EGLNs* and *HIF1A* could influence their expression. Understanding the control of expression levels of *EGLNs* and *HIF1A* in HNSCC pathogenesis may provide insight for potential therapeutic strategies aimed at targeting hypoxia signaling pathways to improve patient outcomes.

Hence, we aimed to assess the expression levels of prolyl hydroxylases (*EGLNs*), *HIF1A*, and selected hypoxia-related genes (*VEGF*, *CA9*, *GLUT1*) in tissues obtained from HNSCC patients and determine the role of DNA methylation in their expression regulation. To the best of our knowledge, this study describes, for the first time, the expression patterns of all proline hydroxylases in tumor and normal tissues of HNSCC patients and correlates these differences with DNA methylation and patients’ clinical characteristics.

## 2. Results

### 2.1. Expression Analysis of HIF1A and EGLNs in HNSCC Based on TCGA Data

**Figure 1:**
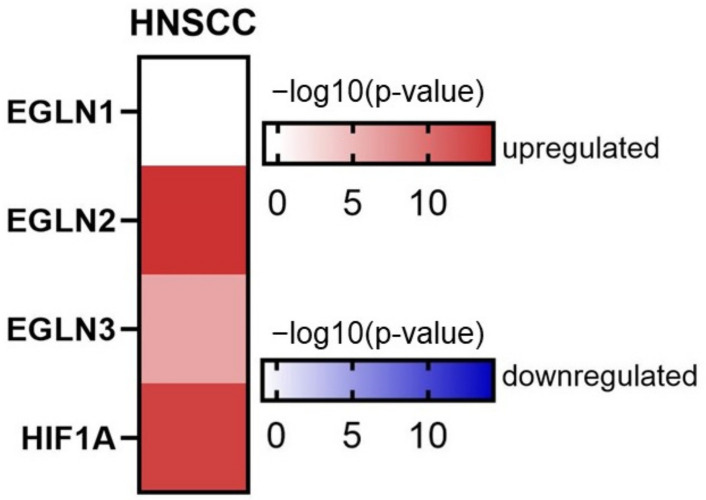
Expression analysis of *EGLNs* and *HIF1A* in tumor and normal tissues based on TCGA data. The heatmap presents log10-transformed statistical significance (*p*-value). Color on the heatmap denotes either upregulated (red) or downregulated (blue) expression in tumor tissues. Accessed on 16 May 2024 (*n* tumor tissue: 519, *n* normal tissues = 44).

We analyzed available TCGA data for the expression patterns of *HIF1A* and *EGLN* family members. Specifically, in HNSCC tumors, *EGLN2*, *EGLN3*, and *HIF1A* were significantly upregulated compared to adjacent normal tissues (Figure 1).

### 2.2. Expression of HIF1A and EGLNs in HNSCC Patients Correlates with Clinicopathological Features and Overall Survival

**Figure 2:**
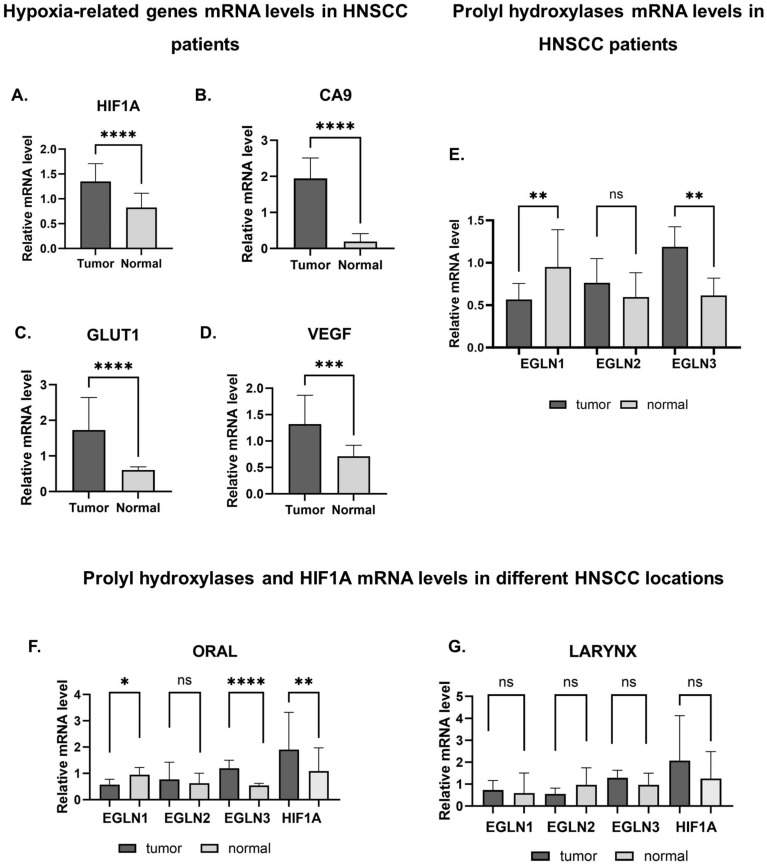
The relative mRNA levels of hypoxia-related genes ((**A**) *HIF1A*, (**B**) *CA*9, (**C**) *GLUT*1, (**D**) *VEGF*) and (**E**) prolyl hydroxylases (*EGLN1*, *EGLN2*, *EGLN3)* in 96 paired samples from HNSCC patients. Each sample was analyzed in triplicate and normalized to the geometric mean of *PBGD* and *SDHA* cDNA levels. The horizontal lines represent the median. The *p*-value was evaluated by the Mann–Whitney U-test. The relative gene expression of prolyl hydroxylases and *HIF1A* was separately evaluated in the oral cavity (**F**) and larynx (**G**) locations of HNSCC. Statistical significance was determined using the Mann–Whitney U-test. *p* ≤ 0.05 is marked as *, *p* ≤ 0.01 is marked as **, *p* ≤ 0.001 is marked as ***, *p* ≤ 0.0001 is marked as ****, and ns is marked as not significant.

We assessed the hypoxia-related gene expression (*HIF1A*, *EGLN1-3*, *CA9*, *GLUT1*, *VEGF*) by qPCR in our study group of 96 tumor tissues and 96 adjacent normal tissues of HNSCC patients (Figure 2A–D). The *HIF1A* expression was significantly higher in tumor tissues compared to normal ones (*p* < 0.0001) (Figure 2A). Moreover, we observed significantly higher mRNA levels of hypoxia-related downstream genes in tumors than in normal tissues (*CA9*, *p* < 0.001; *VEGF*, *p* = 0.0004; and *GLUT1*, *p* < 0.0001) (Figure 2B–D). Subsequently, we evaluated the mRNA levels of all three hydroxylases (*EGLN1-3*). While we did not detect a significant difference in *EGLN2* mRNA levels (Figure 2E), *EGLN1* mRNA levels were significantly higher in normal tissues compared to tumors (*p* = 0.0046) (Figure 2E). Furthermore, the relative mRNA level of *EGLN3* in primary tumors of HNSCC patients was significantly higher than in normal tissues (*p* = 0.0026) (Figure 2E). Subsequently, we also assessed mRNA levels separately in different tumor locations, namely the oral cavity and larynx. The oral cavity tumor results reflected data without stratification, namely, we observed a significant decrease in *EGLN1* levels compared to normal tissues (*p* = 0.0032), while *EGLN3* and *HIF1A* mRNA levels were higher (*p* < 0.0001; *p* = 0.0012) (Figure 2F). However, we did not find significant differences in tumors located in the larynx for any of the *EGLN* genes (Figure 2G). Since PHD2 (EGLN1) demonstrates the highest efficiency in hydroxylating the HIF1A subunit compared to EGLN2 and EGLN3, which are more active towards HIF2A, we focused on the assessment of EGLN1 protein levels within our patient cohort. We observed no statistically significant differences in EGLN1 protein levels between the examined tissues (Supplementary File S2).

Moreover, we observed significantly lower *EGLN*1 mRNA levels in tumor tissues in both age groups (above and under 60 years old), female patients, and different T and N tumor stages and grades. We also found the upregulation of *EGLN1* in normal tissue in the oral cavity (Table 1). We did not find any differences in *EGLN2* expression patterns and clinical features of HNSCC patients (Supplementary File S3). The *EGLN3* mRNA levels were significantly different in tumor tissues in both age groups, in male patients, in different tumor stages and grades, and, in the oral cavity tumor location. Furthermore, *HIF1A* upregulation in tumor tissue was found in the oral cavity tumor location, in male patients, in different TNM stages, and in a group of patients over age 60 (Table 1).

**Figure 3:**
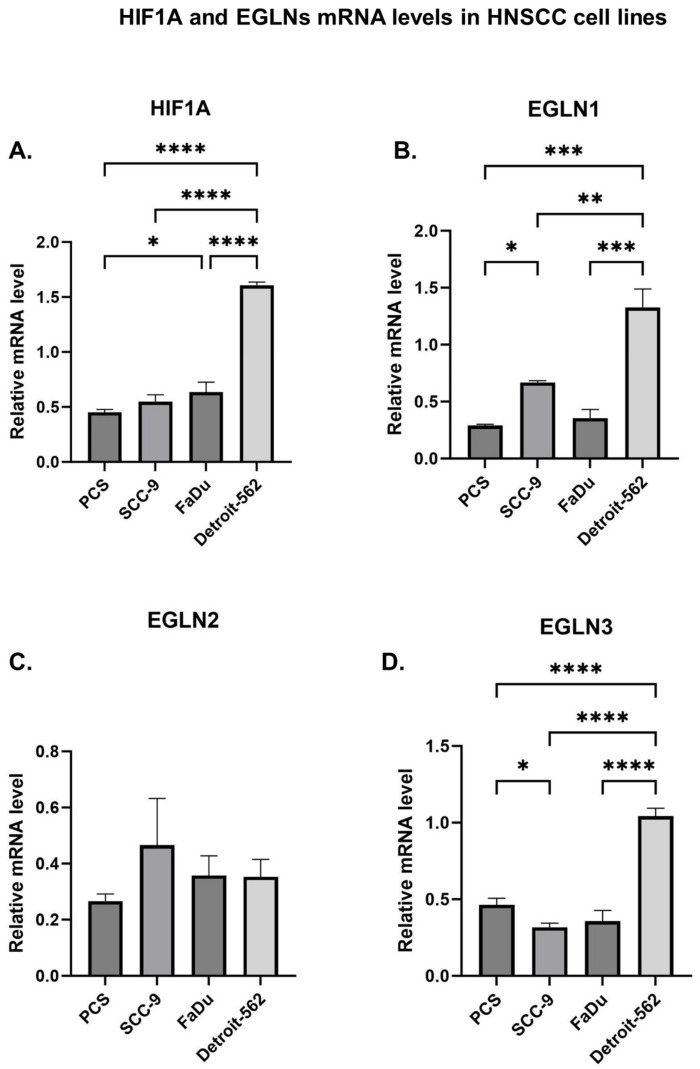
Relative mRNA levels of (**A**) *HIF1A* and prolyl hydroxylases: (**B**) *EGLN1*, (**C**) *EGLN2*, and (**D**) *EGLN3* were assessed in HNSCC cell lines and primary epidermal keratinocytes (PCS). The *p*-value was evaluated by one-way ANOVA test. *p* ≤ 0.05 is marked as *, *p* ≤ 0.01 is marked as **, *p* ≤ 0.001 is marked as ***, *p* ≤ 0.0001 is marked as ****, and ns is marked as not significant.

We also established the expression levels of investigated genes in HNSCC cell lines and primary epidermal keratinocytes, as a healthy control. SCC-9 and FaDu correspond with the oral cavity location (tongue and hypopharynx, respectively) and Detroit-562 is derived from the pleural fluid of an adult female with primary pharynx carcinoma. Interestingly, we observed significantly higher mRNA levels of *HIF1A*, *EGLN1*, and *EGLN3* in the metastatic Detroit-562 cell line compared to both non-metastatic SCC-9 (*p* < 0.0001, *p* = 0.0033, *p* < 00001) and FaDu (*p* < 0.0001, *p* = 0.0003, *p* < 0.0001) cell lines (Figure 3), as well as to the control cell line (*p* < 00001; *p* < 0001; *p* < 00001). We did not observe any differences in *EGLN2* mRNA levels between the examined cell lines.

**Figure 4:**
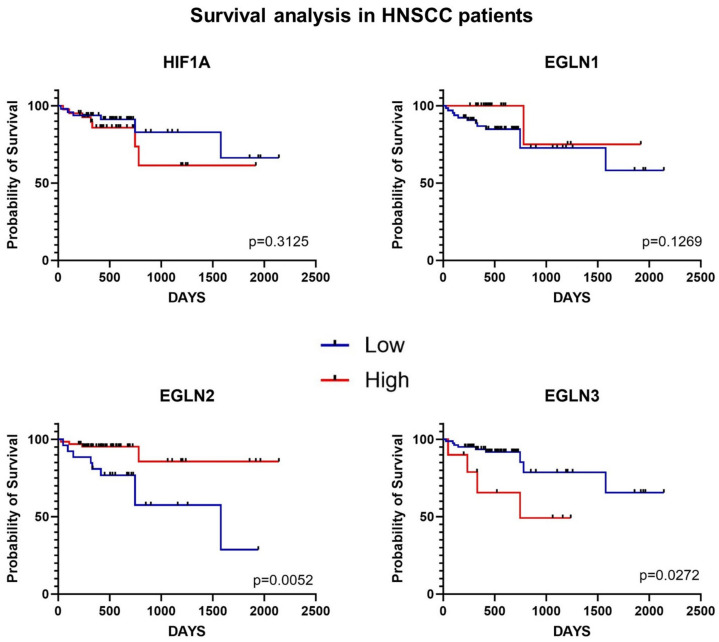
The Kaplan–Meier overall survival analysis among patients with HNSCC according to the optimal gene expression cutoff. Survival time is demonstrated in days.

Subsequently, we performed survival analysis using the Kaplan–Meier method to explore the impact of *EGLN* and *HIF1A* mRNA levels in tumor tissues on patients’ overall survival. Transcript levels of *EGLN1*, *EGLN2*, *EGLN3*, and HIF1A were categorized into high and low expression groups using optimal cutoff points determined by the Cutoff Finder application. Our analysis revealed a significant increase in overall survival (OS) among patients with high *EGLN2* mRNA levels in tumor tissues (*p* = 0.0052) (Figure 4), alongside those with low levels of *EGLN3* (*p* = 0.0272) (Figure 4).

### 2.3. DNA Methylation Analysis

#### DNA Methylation in Promoter Regions of HIF1A and EGLN1-3 in Head and Neck Squamous Cell Carcinoma Patients

**Figure 5:**
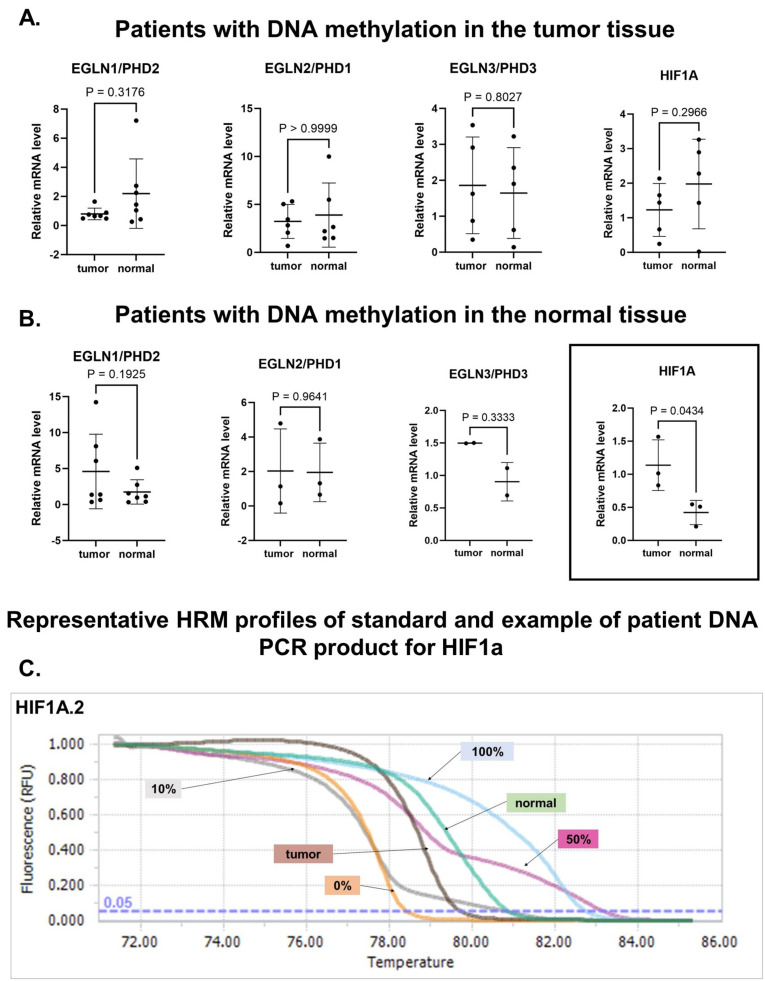
Significance of DNA methylation level in promoter regions assessed with HRM analysis of transcript levels of *HIF1A* and prolyl hydroxylase (*EGLN1-3*) genes in HNSCC patients with hypermethylation in tumor (**A**) or normal tissues (**B**). The statistically significant results was framed. Bisulfite conversion of DNA and qPCR was performed before HRM analysis. The differences between tissue methylation status and the gene expression level were estimated with the Mann–Whitney test. The horizontal line represents the median value. A *p*-value < 0.05 was considered statistically significant. (**C**) Representative result of HRM profile of HNSCC patient.

To determine the DNA methylation status of *EGLN1-3* and *HIF1A* promoter regions in both tumor and normal tissues in our patient cohort (*n* = 77), we used methylation-sensitive High-Resolution Melting Analysis (MS-HRM) (Figure 5A,B). HRM analysis of bisulfite-converted DNA allowed for the detection of differences in DNA sequences based on the melting curve (Figure 5C). In our study group, we found DNA hypermethylation of selected gene promoter regions in tumor or normal tissues for small subgroups of patients (Figure 5). In a subgroup of patients (*n* = 3), we found DNA hypermethylation of the *HIF1A* promoter region in normal tissues compared to tumors, which was associated with a reduced *HIF1A* mRNA level in normal tissues (*p* = 0.04) (Figure 5B). We have presented the clinicopathological features of the patient groups with hypermethylation of *HIF1A* in normal tissues in Table 2, indicating its occurrence in male patients above 60 years old and tumors located in the oral cavity with grade 2 but differences in T stage and N stage. The DNA methylation alterations found in other investigated genes unfortunately did not reflect the changes in mRNA expression level (Figure 5).

To investigate potential differences in DNA methylation between tumor and adjacent normal tissues based on TCGA data, we utilized the UALCAN database to visualize promoter DNA methylation status in *HIF1A* and *EGLN1-3* genes among HNSCC patients (Supplementary File S3). We observed a higher DNA methylation level of the *HIF1A* promoter region in normal tissues compared to tumors (*p* = 0.002) and, on the contrary, higher DNA methylation in promoter regions of *EGLN2* and *EGLN3* (*p* = 0.04 and *p* < 0.0001) in HNSCC tumor tissues. Furthermore, employing TCGA data obtained from cBioportal, we conducted Spearman correlation analyses to evaluate the association between transcript levels of *EGLN1-3* and *HIF1A* and DNA methylation (Supplementary File S4).

## 3. Discussion

Our study aimed to assess hypoxia-associated gene expression in tumors and normal tissues of 96 HNSCC patients, alongside examining the DNA methylation of the promoter region. Additionally, we aimed to characterize the hypoxic effect within our study cohort by evaluating mRNA levels not only of *HIF1A* but also its downstream genes *GLUT1*, *CA9*, and *VEGF* [7,16].

Firstly, our results support previous studies regarding the presence of hypoxia-associated genes in head and neck tumors [3,17]. The mRNA expression of *HIF1A*—the main transcriptional regulator of hypoxia—is modulated by the prolyl hydroxylase family (PHDs). Expression analysis based on TCGA data revealed differential expression patterns of prolyl hydroxylases and HIF1A that varied across tumor types. The survival characteristics of patient groups were influenced by the up- or downregulation of these genes. Our analysis by RT-qPCR of the HNSCC cohort demonstrated the upregulation of *EGLN1* and downregulation of *EGLN3* and *HIF1A* mRNA levels in normal tissue compared to tumor ones, but no significant differences in *EGLN2* levels. Interestingly, we demonstrated the same results when focusing on the oral cavity tumor location, but we did not find any differences in gene expression in the larynx tumors.

HIF1A is overexpressed in various tumors, including colon, breast, lung, and prostate cancer [18,19,20]. Bioinformatical analysis reveals that *HIF1A* is correlated with the increased tumor immune signature and more aggressive tumor phenotypes [21]. Similarly, HNSCC tumors exhibit higher HIF1A expression both in mRNA and protein levels. It was proven that high expression of HIF1A is related to poor outcomes in oropharynx and larynx tumors [22]. It is also interesting and proves our results that HIF1A expression appears to be enhanced in HNSCC cell lines derived from metastatic tumor sites [23].

Similar to our results, other studies indicate a decreased *EGLN1* mRNA level in non-small-cell lung cancer compared to normal lung tissues and colorectal cancer [11,12]. Additionally, low *EGLN1* expression is also correlated with high-grade tumors and poor overall survival of CRC patients [11,24]. Moreover, EGLN1 overexpression may function as a tumor suppressor in pancreatic cancer, which was observed in pancreatic mouse models with decreased tumor growth [25]. Meanwhile, data regarding HNSCC are not conclusive. In nasopharyngeal carcinoma, contrary to our results, the level of IHC-stained EGLN1 was detected to be higher compared to that in normal tissues, with its expression correlating with larger tumor size [26]. Lukkuuaa et al. found low expression levels of EGLN1 protein in normal tissues and well-differentiated head and neck tumors and correlated these data with favorable radiotherapy responses [27]. However, in our analysis, we did not detect significant differences between tumor and normal tissues at the *EGLN1* protein level, probably due to the limited sample size.

In colorectal cancer, the mRNA and protein levels of EGLN2 were lower in the primary cancer than in histopathologically unchanged tissues [11]. Similarly, a decreased mRNA level of *EGLN2* was found in non-small-cell lung cancer and was correlated with larger tumors and higher tumor stage [12]. On the contrary, the mRNA level of *EGLN2* was upregulated in pancreatic cancer compared to matched normal pancreatic regions [28]. We did not find any differences in the mRNA level of *EGLN2* in tumors versus normal HNSCC tissues, nor between HNSCC cell lines. 

We demonstrated significant upregulation of *EGLN3* mRNA levels in tumors compared to normal tissues from HNSCC patients. Also, Högel et al. showed an increased mRNA level of *EGLN3* in HNSCC cell lines and its association with hypoxia markers. Furthermore, the inhibition of EGLN3 decreased cell survival under hypoxic conditions [29]. Similarly, in hepatocellular carcinoma (HCC), the EGLN3 mRNA and protein levels were significantly higher in tumor tissues compared to adjacent non-tumor liver samples [30]. The bioinformatics analysis demonstrated that *EGLN3* expression was higher in lung adenocarcinoma (LUAD) compared to adjacent normal tissues and its high level was positively associated with tumor purity, histological type, higher stage, as well as poor prognosis [31]. Decreased *EGLN3* expression was detected in prostate, breast, melanoma, and renal carcinoma cell lines [32]. Similarly, gastric cancer exhibits a decrease in the mRNA level of *EGLN3* in cancerous tissues compared to adjacent normal tissues [33]. The possible role of upregulated EGLN3 in solid tumors is complex, influencing cell proliferation and migration, and its expression is tied to various grades and outcomes. For instance, in breast cancer, diminished EGLN3 expression correlates with larger, less-differentiated tumors [34], while in pancreatic cancer, EGLN3 overexpression is associated with well-differentiated tumor occurrence [35]. Moreover, high expression of nuclear forms of EGLN2 and EGLN3 was associated with worse survival in pancreatic endocrine cancer [36]. The interplay between cell type and oxygen availability emerges as pivotal in understanding the dualistic function of EGLN3 [29]. EGLN3 plays a crucial role in modulating hypoxia-related genes, including numerous glycolytic enzymes, thereby influencing lactate production [37]. Reduction of EGLN3 expression in tumor cells suppresses EGFR internalization, resulting in its hyperactivation [38]. Recent studies highlight that EGLN3 can shield cancer cells from death induced either by hypoxia or other apoptotic factors [39]. Under hypoxia, EGLN3 hydroxylates PKM2, subsequently facilitating its interaction with HIF1A in the nucleus, enhancing its activity [38,40]. Notably, inhibition of EGLN3 causes cell cycle arrest of HNSCC cells under hypoxic conditions [29].

The gene expression analysis conducted on HNSCC cell lines intriguingly revealed distinctions between metastatic (Detroit-562) and non-metastatic cell lines (FaDu, SCC-9). Our findings indicate elevated mRNA levels of *HIF1A*, *EGLN1*, and *EGLN3* in metastatic Detroit-562 cells compared to FaDu and SCC-9 cell lines.

The clinicopathological characteristics of HNSCC patients revealed that OS-related results for *EGLN1* did not present a beneficial impact of low *EGLN1* transcript level, but we observed a significant increase in OS among patients with high *EGLN2* mRNA levels in tumor tissues (*p* = 0.0052), as well as those with low levels of *EGLN3* (*p* = 0.0272). Similar to our results, in non-small-cell lung cancer, a higher mRNA level of *EGLN2* contributes to longer overall survival. Notably, in pancreatic cancer, decreased expression of EGLN2 and EGLN3 resulted in the induction of angiogenic factors by HIF1A and TGF-β1 pathway and poor patient OS [28].

Our findings revealed significant variations in *EGLN* expression patterns across different tumor types, impacting patients’ survival in distinct manners. The *EGLN* gene family exerts an influence on the tumor microenvironment through the regulation of hypoxia-inducible factor expression. Additionally, epigenetic DNA modifications can also regulate HIF1A.

The expression of *EGLN* in cancer is context-dependent and can have diverse implications for tumor behavior and patient outcomes, and yet the mechanisms of its altered expression are still not fully elucidated. Abnormal DNA methylation patterns frequently occur in various cancers and may contribute to tumorigenesis, but they also could constitute markers to distinguish between tumor and normal tissues [41].

In breast cancer, DNA hypomethylation of the promoter region of *HIF1A* was found in breast cancer epithelial cells with highly malignant biological behavior [42]. On the contrary, in uterine cervical carcinoma, there were no significant DNA methylation changes in either control or cancerous tissue samples [43].

Our results suggest that HNSCC patients with hypermethylation in the *HIF1A* promoter region in normal tissues display lower mRNA levels of this gene in normal tissues compared to hypomethylated tumor ones. However, these findings should be interpreted with caution due to potential bias arising from the sample size, and the study should be extended to a larger group of patients to identify its biological significance and prognostic value. The analyses of biological databases support our result, indicating the higher DNA methylation level of the *HIF1A* promoter region in normal tissues of HNSCC compared to tumors, in which expression is significantly higher. However, our HRM-based analysis did not confirm the influence of DNA methylation on differences between mRNA levels of *EGLN* genes in tumors versus normal tissues. Still, the TCGA-obtained data suggest a potential negative correlation between transcript levels of *EGLN2* and *EGLN3* and DNA methylation of their promoter region, as well the impact of this DNA methylation on HNSCC patients’ overall survival. The lack of confirmation might arise from the limited sample size as well as not detecting DNA methylation in the limited bp range of the HRM-qPCR amplicons. Nevertheless, few studies point out the role of DNA methylation in EGLN expression regulation. Lower DNA methylation of CpG sites in *EGLN1* correlated with upregulated plasma EGLN1 levels in high-altitude pulmonary edema [44]. However, DNA methylation in the CpG island of the *EGLN3* promoter region caused reduced expression of this gene in colorectal cancer and, similarly, in leukemic cell lines with decreased mRNA and protein expression [45]. Furthermore, the *EGLN3* promoter region was found to be hypermethylated in prostate, breast, melanoma, and renal carcinoma cell lines [32].

In summary, our findings demonstrate that mRNA levels of *HIF1A* and hypoxia-related genes (*CA9, VEGF, GLUT1*) are increased in the tumor tissues of HNSCC patients compared to normal tissues. Moreover, *EGLN3* mRNA levels are elevated in HNSCC tumors, whereas *EGLN1* is downregulated. Notably, *HIF1A* and *EGLN3* mRNA levels show an upregulation in metastatic HNSCC cell lines when compared to cell lines originating from oral cancer. Additionally, the presence of DNA methylation within the *HIF1A* promoter region in normal tissues of HNSCC patients may contribute to the regulation of its expression. Nonetheless, further large-scale studies are imperative to validate clinical relevance and explore potential future implications.

## 4. Materials and Methods

### 4.1. Antibodies and Reagents

Rabbit polyclonal (Rp) anti-PHD2 (PA5-17050) was purchased from Thermo Fisher Scientific (Waltham, MA, USA). Rp anti-β-tubulin HRP-conjugated Ab (ab21058) was purchased from Abcam (Abcam, Cambridge, UK).

### 4.2. Clinical Material

Primary tumors and paired-matched normal tissues were collected from 96 patients with HNSCC who underwent tumor surgical resection in the Department of Head and Neck Surgery, Poznan University of Medical Sciences, The Greater Poland Cancer Center. Samples were immediately snap-frozen in liquid nitrogen and stored at −80 °C until RNA, DNA, and protein isolation. The characterization of the total study cohort is presented in Table 3. Exclusion criteria included distant metastases and second primary tumors. The procedures were approved by the Local Ethical Committee of Poznan University of Medical Sciences (Protocol code 452/20, date of approval 17 June 2020).

### 4.3. Cell Culture

The FaDu, Detroit-562, SCC-9, and primary epidermal keratinocyte normal, human, adult (PCS-200-011) cell lines were obtained from the American Type Culture Collection (ATCC™, Manassas, VA, USA). The FaDu cells were cultured in Dulbecco’s modified Eagle’s Medium (DMEM) (Biowest, Nuaillé, France), the Detroit-562 cells in Eagle’s Minimum Essential Medium (EMEM) (Biowest, Nuaillé, France), the SCC-9 cells in a 1:1 mixture of DMEM and Ham’s F12 Medium (Biowest, Nuaillé, France), and the PCS-200-011 cells in Dermal Cell Basal Medium with Keratinocyte Growth Kit. All growth media were supplemented with 10% fetal bovine serum (FBS, Biowest, Nuaillé, France) and 1% penicillin/streptomycin (Biochrom, Holliston, MA, USA). The cell lines were cultured in an incubator at 37 °C, in a 5% CO_2_ atmosphere, and at a humidity level of 100%.

### 4.4. RNA Isolation, Reverse Transcription, and Real-Time Quantitative Polymerase Chain Reaction (RT-qPCR) Analysis

Total RNA was isolated from tissues and cell lines using an RNA purification kit (RNeasy Mini Kit, Qiagen, Hilden, Germany). RNA samples were quantified by spectrometric measurement and qualified by gel electrophoresis. Subsequently, the samples were reverse-transcribed into cDNA with the RevertAid First Strand cDNA Synthesis Kit (Thermo Fisher, Waltham, MA, USA), using 500 ng of total RNA. RT-qPCR was carried out with the CFX96 Real-Time System (Bio-Rad, Hercules, CA, USA) using PowerTrack SYBR Green Master Mix (Thermo Fisher, Waltham, MA, USA). The gene expression level was normalized to the geometric mean of two internal controls SDHA (succinate dehydrogenase complex flavoprotein subunit A) and PBGD (porphobilinogen deaminase) genes, and the relative expression level was determined by the Pfaffl method. The calibrator was prepared as a mixture of the patients’ cDNA and successive dilutions were used to create a standard curve to evaluate the efficiency rate of each primer. The primer sequences used in this study are listed in Supplementary File S1.

### 4.5. DNA Isolation, Bisulfite Conversion, and Methylation-Sensitive High-Resolution Melting (MS-HRM)

Genomic DNA from tumors and normal tissues was extracted using the DNA Mammalian Genomic Purification Kit from Sigma-Aldrich Co., (St. Louis, MO, USA). The EZ DNA Methylation Kit™ was used to carry out the bisulfite conversion with 500 ng of isolated DNA (Zymo Research Corporation, Irvine, CA, USA). To assess the DNA methylation levels of CpG islands in the promoter regions of analyzed genes, Real-Time PCR amplification of bisulfite-treated DNA followed by HRM profile analysis was carried out using the LightCycler^®^480 Real-Time PCR System (Roche Diagnostics GmbH, Mannheim, Germany). The PCR mixture included converted DNA, primers, and 5X Hot FIREPol EvaGreen HRM Mix (Solis BioDyne Co. Tartu, Estonia). To determine the percentage of methylation, the HRM profiles of patient DNA PCR products were compared with HRM profiles of standard DNA PCR products, prepared by mixing methylated and unmethylated bisulfite-treated DNA from the Human Methylated/Unmethylated DNA Set (Zymo Research Corp., Orange, CA, USA). For statistical analysis, the percentage results were divided into 0–10% methylation, 10–50% methylation, and 50–100% methylation based on the method used by Rawluszko et al. [11]. Moreover, the CpG distribution in regulatory regions of PHDs is also available in this author’s previous work [11]. The primer sequences used in this study are listed in Supplementary File S1 and the representative HRM profiles of all genes are shown in Supplementary File S5.

### 4.6. Western Blot Analysis

Protein isolation was performed in RIPA buffer with protein inhibitors. The samples were separated by SDS-PAGE to quantify the selected protein level using Mini-PROTEAN TGX precast gels (Bio-Rad, Hercules, CA, USA). Subsequently, the gel was transferred to a PVDF membrane using Trans-Blot Turbo transfer packs (Bio-Rad, Hercules, CA, USA) and blocked with 5% milk in TBST buffer. Immunodetection of bands was performed with Rp anti-PHD2, followed by incubation with goat anti-rabbit HRP-conjugated Ab. Rp anti-beta-tubulin Ab was used to detect the reference protein. Bands were revealed using Clarity Western ECL Blotting Substrate (Bio-Rad, Hercules, CA, USA) and the ChemiDoc™ Touch Imaging System (Bio-Rad, Hercules, CA, USA).

### 4.7. Bioinformatical Analysis

#### 4.7.1. The Gene Expression in TCGA Solid Tumors

The expression of *HIF1A* and *EGLN1-3* genes in tumors and normal tissues of the HNSCC TCGA tumor cohort was analyzed using the Gene_DE module of the TIMER2.0 platform (http://timer.cistrome.org/) [46]. The Gene_DE module allows users to study the differential expression (log2-normalized TPM values) between tumor and adjacent normal tissues for any gene of interest across TCGA tumors. The statistical significance was computed by the Wilcoxon test. Accessed on 16 May 2024 (*n* tumor tissues: 519, *n* normal tissues = 44).

#### 4.7.2. Transcriptomic and DNA Methylation Data

The RNA sequencing-based mRNA expression and DNA methylation data were directly downloaded from the cBioportal (www.cbioportal.org) [47]. RNASeq V2 from TCGA was processed and normalized using RSEM. The DNA methylation data were downloaded as Methylation (HM450) beta-values. The Spearman correlation between these two variables was estimated using the GraphPad Prism 10 software. Accessed on 1 May 2024 (*n* tumor tissues: 519, *n* normal tissues = 44).

### 4.8. Statistical Analysis

The normality of the observed patients’ data distribution was assessed using the Shapiro–Wilk test. The median values were compared using the Mann–Whitney test, and the mean values were compared using an unpaired *t*-test. The differences between more than two groups were estimated with one-way ANOVA. The correlation between the studied variables was determined using Spearman’s rank correlation. Statistical analysis was performed using GraphPad Prism 10 software, and *p* < 0.05 was considered statistically significant. The patient survival analyses were estimated using the Kaplan–Meier method. The optimal cutoff points of expression differentiating patients based on survival were determined using the Cutoff Finder application [48]. In all graphs, *p* ≤ 0.05 is marked as *, *p* ≤ 0.01 is marked as **, *p* ≤ 0.001 is marked as ***, *p* ≤ 0.0001 is marked as ****, and ns is marked as not significant.

## 5. Conclusions

To conclude, we demonstrate that EGLN and hypoxia-related genes are differentially expressed in HNSCC tissues, and their level depends on the clinical characteristics of patients. DNA methylation emerges as a potential mechanism driving alterations in the *HIF1A* gene among HNSCC patients. Additionally, we found that levels of prolyl hydroxylases and *HIF1A* are associated with HNSCC patients’ overall survival, highlighting their role in cancer progression.

## Figures and Tables

**Table 1:** Gene transcript levels in tumor and normal tissue samples from patients with HNSCC.

	*EGLN1*		*EGLN3*		*HIF1A*	
	Mean/Median	*p*-Value	Mean/Median	*p*-Value	Mean/Median	*p*-Value
Age ≤ 60	tumor = 0.52 normal = 1.05	0.001	tumor = 1.06 normal = 0.47	0.014	tumor = 1.31 normal = 1.09	ns
Age > 60	tumor = 0.59 normal = 0.95	0.039	tumor = 1.31 normal = 0.66	0.006	tumor = 1.35 normal = 0.67	<0.0001
Male	tumor = 0.57 normal = 0.89	ns	tumor = 1.27 normal = 0.62	0.004	tumor = 1.35 normal = 0.69	<0.0001
Female	tumor = 0.59 normal = 0.95	0.023	tumor = 0.84 normal = 0.45	ns	tumor = 1.35 normal = 1.00	ns
T1–T2	tumor = 0.61 normal = 1.23	ns	tumor = 0.67 normal = 0.42	ns	tumor = 1.34 normal = 0.70	0.019
T3	tumor = 0.50 normal = 0.87	ns	tumor = 1.47 normal = 0.69	0.002	tumor = 1.65 normal = 1.24	0.024
T4	tumor = 0.57 normal = 0.93	0.018	tumor = 0.90 normal = 0.62	ns	tumor = 1.36 normal = 0.81	0.018
N0	tumor = 0.47 normal = 0.93	0.011	tumor = 1.30 normal = 0.87	ns	tumor = 1.11 normal = 0.67	0.036
N1	tumor = 0.72 normal = 1.64	0.029	tumor = 1.33 normal = 0.55	0.002	tumor = 1.90 normal = 0.94	0.023
N2	tumor = 0.60 normal = 0.89	ns	tumor = 1.41 normal = 0.62	0.018	tumor = 1.65 normal = 1.09	ns
G2	tumor = 0.51 normal = 0.85	0.037	tumor = 1.08 normal = 0.66	0.039	tumor = 1.35 normal = 0.81	0.0003
G3	tumor = 0.68 normal = 1.78	0.042	tumor = 1.78 normal = 0.48	ns	tumor = 1.64 normal = 1.04	ns
Oral cavity	tumor = 0.57 normal = 0.95	0.032	tumor = 1.20 normal = 0.54	< 0.0001	tumor = 1.50 normal = 0.87	0.001

Gene transcript levels were measured in triplicate and standardized by *PBGD* and *SDHA* reference genes, relative gene expression was calculated using the Pfaffl method. We performed the Mann–Whitney U-test or unpaired *t*-test based on the normality data distribution. ns—nonsignificant.

**Table 2:** Clinicopathological characteristics of HNSCC patients with *HIF1A* DNA hypermethylation in normal tissues.

Characteristics	No. of Cases with *HIF1A* DNA Hypermethylation in Normal Tissues
Age (<60/>60)	0/3
Gender (female/male)	0/3
Localization (oral/larynx)	3/0
Histological grade (G1/G2/G3)	0/3/0
TNM (I/II/III/IV)	0/1/2/0
N stage (0/1/2/3)	1/0/1/1

**Table 3:** Characteristics of the study cohort.

Characteristic	Total Number (*n*/%)
Patient study cohort	96
Age at the time of surgery (years)	
Mean	63.48
Median	64
Range	36–91
Gender [*n*/(%)]	
Male	73 (76%)
Female	23 (24%)
Tumor stage (TNM classification) [*n*/(%)]	
T1	4 (4%)
T2	21 (22%)
T3	30 (31%)
T4	41 (43%)
N0	38 (40%)
N1	21 (22%)
N2	25 (26%)
N3	9 (9%)
Histologic grade [*n*/(%)]	
G1	16 (17%)
G2	66 (69%)
G3	14 (15%)
Tumor location [*n*/(%)]	
Larynx	38 (40%)
Oral cavity	58 (60%)

## Data Availability

Publicly available data were used in this study, and data sources and handling of these data are described in the Section 4. Further information is available from the corresponding author upon request.

## Supplementary Materials

The following supporting information can be downloaded at: https://www.mdpi.com/article/10.3390/ijms25126495/s1.
