# Supplementary material for: Understanding Hypoxia-Driven Tumorigenesis: The Interplay of HIF1A, DNA Methylation, and Prolyl Hydroxylases in Head and Neck Squamous Cell Carcinoma
Source: Int J Mol Sci. 2024 Jun 12;25(12):6495. doi: 10.3390/ijms25126495 (PMC11203966; doi:10.3390/ijms25126495)
Supplement: Supplementary file 1 [file ijms-25-06495-s001.zip › Supplementary file S1.pdf]

Table S1. Primers sequences.

Gene	Sequence (5’-3’)	Product length (bp)	Method
PHD1	F: CCAAGGTAAGGCTAGGTGGG<br>R: ATTAAGGCCCGTAGGGAGGA	194	RT-PCR
PHD2	F: CCTGCGACTGAAACTGCCTA<br>R: GTGAACGGGCAGTAAGTGGA	164	RT-PCR
PHD3	F: CCTCTTACGCAACCAGATATG<br>R:AGCACGGTCAGTCTTCAG	129	RT-PCR
HIF1A	F: AGAGGTTGAGGGACGGAGAT<br>R: CAACATGAAATGTCCTGCGT	200	RT-PCR
PBGD	F: GCCAAGGACCAGGACATC<br>R: TCAGGTACAGTTGCCCATC	160	RT-PCR
SDHA	F: GTGCCTGAGAACGAATGGAGA<br>R: TGACAGGTGGTGTTCACAGA	302	RT-PCR
CA9	F: GGCTACAGCTGAACTTCCGA<br>R: AATTCAGCTGGACTGGCTCA	114	RT-PCR
VEGF	F: ACATCACCATGCAGATTATGCG<br>R: CTCCAGGGCATTAGACAGCA	229	RT-PCR
PHD1.1	F: GTGTGGTTATGAATTTTGTGTG<br>R: CTACACCAATCCCCTACAAAC	142	MS-HRM
PHD1.2	F: CGGGTAGGTTTGTTTATGTAA<br>R: ACACGAAATTCATTTACATCC	165	MS-HRM
PHD2.1	F: GGGAGTTTGATTTTTGGATTT<br>R: AATCATAACTACTCTTCCTCC	177	MS-HRM

PHD2.2	F: TTAGCGTTAGGATTGGAAGAAG<br>R: ACCTTCCTAAACTCCCGAAC	150	MS-HRM
PHD2.3	F:GGGGTTCGGGAGTTTAGGAAGGTAG<br>R: CCGAAAACTTAACCTTTACTTTTCCCTTAAC	88	MS-HRM
PHD3.1	F: CGTGGTAGTCGTAGGTTTTTG<br>R: TCTCCAAATCCAACCTCATAAT	159	MS-HRM
PHD3.2	F: GTTGGATTTGGAGAAAATTG<br>R: ACCCCGATACAATACAACTAC	141	MS-HRM
PHD3.3	F: TTGTATGTCGATTTTTTAGGGT<br>R: ACCTAAACCCCTTAACGTTAAC	139	MS-HRM
HIF1A.1	F: AGATTAAAGGAAGGGTTTGTTGTTA<br>R: AAATATGATGTATGTTTGGGATTAGGT	171	MS-HRM
HIF1A.2	F: GTTGGGTTTTGATAAGTTATTTGAG<br>R:AAAACAAATCCAAAAATAAAAATAC	127	MS-HRM

RT-qPCR- real-time quantitative polymerase chain reaction **MS-HRM**- Methylation sensitive high resolution melting analysis
