# Supplementary material for: Understanding Hypoxia-Driven Tumorigenesis: The Interplay of HIF1A, DNA Methylation, and Prolyl Hydroxylases in Head and Neck Squamous Cell Carcinoma
Source: Int J Mol Sci. 2024 Jun 12;25(12):6495. doi: 10.3390/ijms25126495 (PMC11203966; doi:10.3390/ijms25126495)
Supplement: Supplementary file 1 [file ijms-25-06495-s001.zip › Supplementary file S3. gene expression and patients characteristics.pdf]

Table S2. *EGLN1* transcript level in tumor and normal tissue samples from patients with HNSCC.

Characteristic	Normal tissue	Tumor tissue	*p*-value
	**Median (range*) [a] or Mean (± SD*) [b]**	**Median (range*) [a] or Mean (± SD*) [b]**	
	**Age at the time of surgery (years)**		
≤60	1.05 (0.10-5.6) [a]	0.52 (0.05-1.36) [a]	**0.0014**
>60	0.95 (0.14-4.32) [a]	0.59 (0.11-3.00) [a]	**0.0393**
	**Gender**		
Male	0.89 [a]	0.57 [a]	0.0615
Female	1.14 (0.32-4.12) [a]	0.51 (0.11-2.8) [a]	**0.0229**
	**Tumor stage (TNM classification)**		
T1 – T2	1.23 [a]	0.61 [a]	0.0603
T3	0.87 [a]	0.50 [a]	0.1638
T4	0.93 (0.18-3.44) [a]	0.57 (0.18-1.72) [a]	**0.0183**
N0	0.85 (0.10-4.32) [a]	0.47 (0.05-1.84) [a]	**0.0113**
N1	1.64 (0.32-5.35) [a]	0.72 (0.26-2.99) [a]	**0.0288**
N2	0.89 [a]	0.60 [a]	0.504
N3	0.74 [a]	0.45 [a]	>0.9999
	**Histologic grade**		
G1	1.56 [a]	0.93 [a]	0.3445
G2	0.85 (0.10-4.12) [a]	0.51 (0.05-3.02) [a]	**0.0372**
G3	1.78 (0.14-5.10) [b]	0.68 (0.15-1.36) [b]	**0.0421**
	**Anatomical site**		
Larynx	0.97 [a]	0.55 [a]	0.0578
Oral cavity	0.95 (0.10-4.12) [a]	0.57 (0.05-2.99) [a]	**0.032**

Genes transcript levels were measured in triplicates and standardized by PBGD and SDHA reference genes, relative gene expression was calculated using the Pfaffl method. We performed the U Mann-Whitney testa or unpaired t-test based on the normality data distribution. Range or SD was shown when p-value <0.05.

Table S3. *EGLN2* transcript level in tumor and normal tissue samples from patients with HNSCC.

Characteristic	Normal tissue	Tumor tissue	*p*-value
	Median (range*) [a] or Mean (± SD*) [b]	Median (range*) [a] or Mean (± SD*) [b]	
**Age at the time of surgery (years)**			
≤60	0.58 [a]	0.77 [a]	0.1578
>60	0.62 [a]	0.73 [a]	0.4545
**Gender**			
Male	0.60 [a]	0.77 [a]	0.0802
Female	1.20 [a]	0.66 [a]	0.3226
**Tumor stage (TNM classification)**			
T1-T2	0.93 [a]	0.88 [a]	0.7456
T3	0.75 [a]	1.08 [a]	0.4238
T4	0.61 [a]	0.69 [a]	0.8625
N0	0.93 [a]	0.68 [a]	0.9193
N1	0.54 [a]	0.80 [a]	0.2020
N2	1.01 [a]	1.36 [a]	0.7263
N3	0.79 [b]	0.47 [b]	0.1779
**Histologic grade**			
G1	0.60 [a]	0.77 [a]	0.942
G2	0.62 [a]	0.79 [a]	0.3374
G3	0.96 [a]	0.96 [a]	0.2312
**Anatomical site**			
Larynx	0.60 [a]	0.77 [a]	0.2574
Oral cavity	0.63 [a]	0.77 [a]	0.2756

Genes transcript levels were measured in triplicates and standardized by PBGD and SDHA reference genes, relative gene expression was calculated using Pfaffl method. We performed the U Mann-Whitney testa or unpaired t-testb based on the normality data distribution. Range or SD was shown when p-value <0.05.

Table S4. *EGLN3* transcript level in tumor and normal tissue samples from patients with HNSCC.

Characteristic	Normal tissue	Tumor tissue	*p*-value
	**Median (range*) [a] or Mean (± SD*) [b]**	**Median (range*) [a] or Mean (± SD*) [b]**	
**Age at the time of surgery (years)**			
≤60	0.47 (0.08-1.53) [a]	1.06 (0.0007-3.54) [a]	**0.0143**
>60	0.66 (0.16-2.86) [a]	1.31 (0.07-6.39) [a]	**0.0064**
**Gender**			
Male	0.62 (0.14-2.42) [a]	1.27 (0.14-4.97) [a]	**0.0043**
Female	0.45 [a]	0.84 [a]	0.1350
**Tumor stage (TNM classification)**			
T1-T2	0.42 [a]	0.67 [a]	0.3795
T3	0.69 (0.17-2.42)	1.47 (0.07-5.56)	**0.0016**
T4	0.62 [a]	0.90 [a]	0.1752
N0	0.87 [a]	1.30 [a]	0.1283
N1	0.55 (0.08-2.35) [a]	1.33 (0.14-5.14) [a]	**0.0147**
N2	0.62 (0.20-1.53) [a]	1.41 (0.07-6.26) [a]	**0.018**
N3	0.42 [a]	0.44 [a]	0.6126
**Histologic grade**			
G1	0.53 [a]	1.63 [a]	0.0545
G2	0.66 (0.08-2.42) [a]	1.08 (0.00-4.23) [a]	**0.0389**
G3	0.48 [a]	1.78 [a]	0.106
**Anatomical site**			
Larynx	0.97 [a]	1.29 [a]	0.8385
Oral cavity	0.54 (0.08-1.53) [a]	1.20(0.07-4.68) [a]	**<0.0001**

Genes transcript levels were measured in triplicates and standardized by PBGD and SDHA reference genes, relative gene expression was calculated using Pfaffl method. We performed the U Mann-Whitney testa or unpaired t-testb based on the normality data distribution. Range or SD was shown when p-value <0.05.

Table S5. *HIF1A* transcript level in tumor and normal tissue samples from patients with HNSCC.

Characteristic	Normal tissue	Tumor tissue	*p*-value
	**Median (range*) [a] or Mean (± SD*) [b]**	**Median (range*) [a] or Mean (± SD*) [b]**	
**Age at the time of surgery (years)**			
≤60	1.09 [a]	1.31[a]	0.00565
>60	0.67 (0.02-2.82) [a]	1.35 (0.23-5.07) [a]	**<0.0001**
**Gender**			
Male	0.69(0.02-2.90) [a]	1.35 (0.23-5.07) [a]	**<0.0001**
Female	1.00 [a]	1.35 [a]	0.2637
**Tumor stage (TNM classification)**			
T1-T2	0.70 (0.15-4.00) [a]	1.34 (0.23-5.07) [a]	**0.0189**
T3	1.24 (0.15-4.01) [a]	1.65 (0.32-6.08) [a]	**0.0237**
T4	0.81 (0.02-2.82) [a]	1.36 (0.24-4.92) [a]	**0.0175**
N0	0.67 (0.02-2.18) [a]	1.11 (0.23-3.61) [a]	**0.0364**
N1	0.94 (0.16-2.18) [a]	1.90 (0.29-5.72) [a]	**0.0231**
N2	1.09 [a]	1.65 [a]	0.1130
N3	0.64 [b]	1.93 [b]	0.0581
**Histologic grade**			
G1	1.04 [a]	1.26 [a]	0.4228
G2	0.81 (0.07-3.00) [a]	1.35 (0.23-5.01) [a]	**0.0003**
G3	1.04 [a]	1.64 [a]	0.0763
**Anatomical site**			
Larynx	0.81 (0.02-2.18) [a]	1.18 (0.24-5.58) [a]	**0.0699**
Oral cavity	0.87 (0.07-3.26) [a]	1.50 (0.23-5.72) [a]	**0.0012**

Genes transcript levels were measured in triplicates and standardized by PBGD and SDHA reference genes, relative gene expression was calculated using Pfaffl method. We performed the U Mann-Whitney test[a] or unpaired t-test[b] based on the normality data distribution. Range or SD was shown when p-value <0.05.
