# Supplementary material for: Understanding Hypoxia-Driven Tumorigenesis: The Interplay of HIF1A, DNA Methylation, and Prolyl Hydroxylases in Head and Neck Squamous Cell Carcinoma
Source: Int J Mol Sci. 2024 Jun 12;25(12):6495. doi: 10.3390/ijms25126495 (PMC11203966; doi:10.3390/ijms25126495)
Supplement: Supplementary file 1 [file ijms-25-06495-s001.zip › Supplementary file S4. DNA mathylation TCGA analysis.pdf]

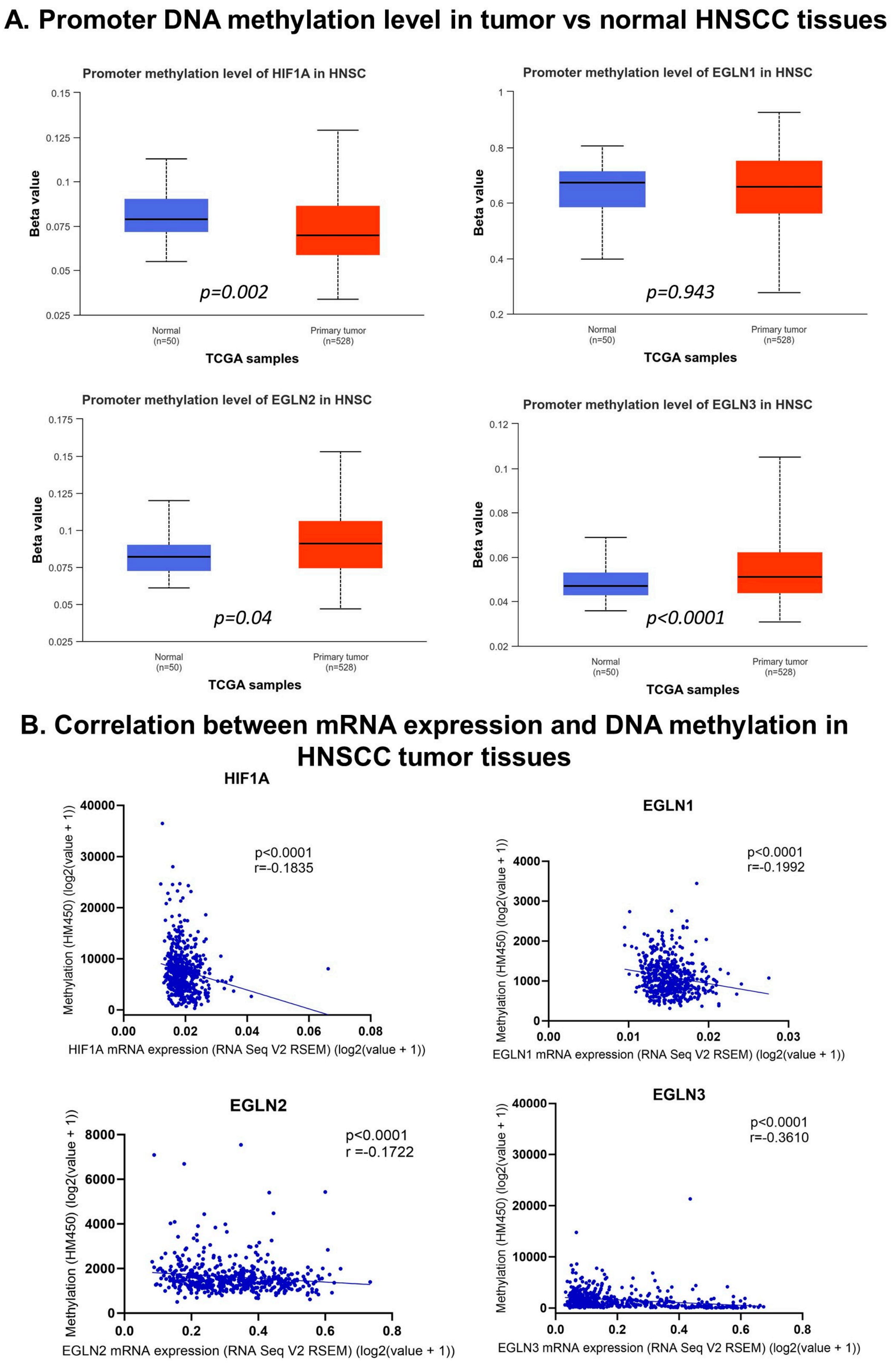

DNA methylation in the HNSCC based on TCGA data. (**A**) DNA methylation in the promoter region of HIF1A and EGLN1-3 genes in tumor versus normal tissues of HNSCC. The Beta value indicates the level of DNA methylation ranging from 0 (unmethylated) to 1 (fully methylated). Graphs were generated using the UALCAN database (https://ualcan.path.uab.edu/index.html). The *P*-value was estimated by Student's t-test considering the unequal variance. (**B**) Spearman correlation between transcript level of *HIF1A* and *EGLN1-3* and DNA methylation performed on data downloaded from cBioPortal (https://www.cbioportal.org/).
