# Supplementary figures and images for: Understanding Hypoxia-Driven Tumorigenesis: The Interplay of HIF1A, DNA Methylation, and Prolyl Hydroxylases in Head and Neck Squamous Cell Carcinoma
Source: Int J Mol Sci. 2024 Jun 12;25(12):6495. doi: 10.3390/ijms25126495 (PMC11203966; doi:10.3390/ijms25126495)

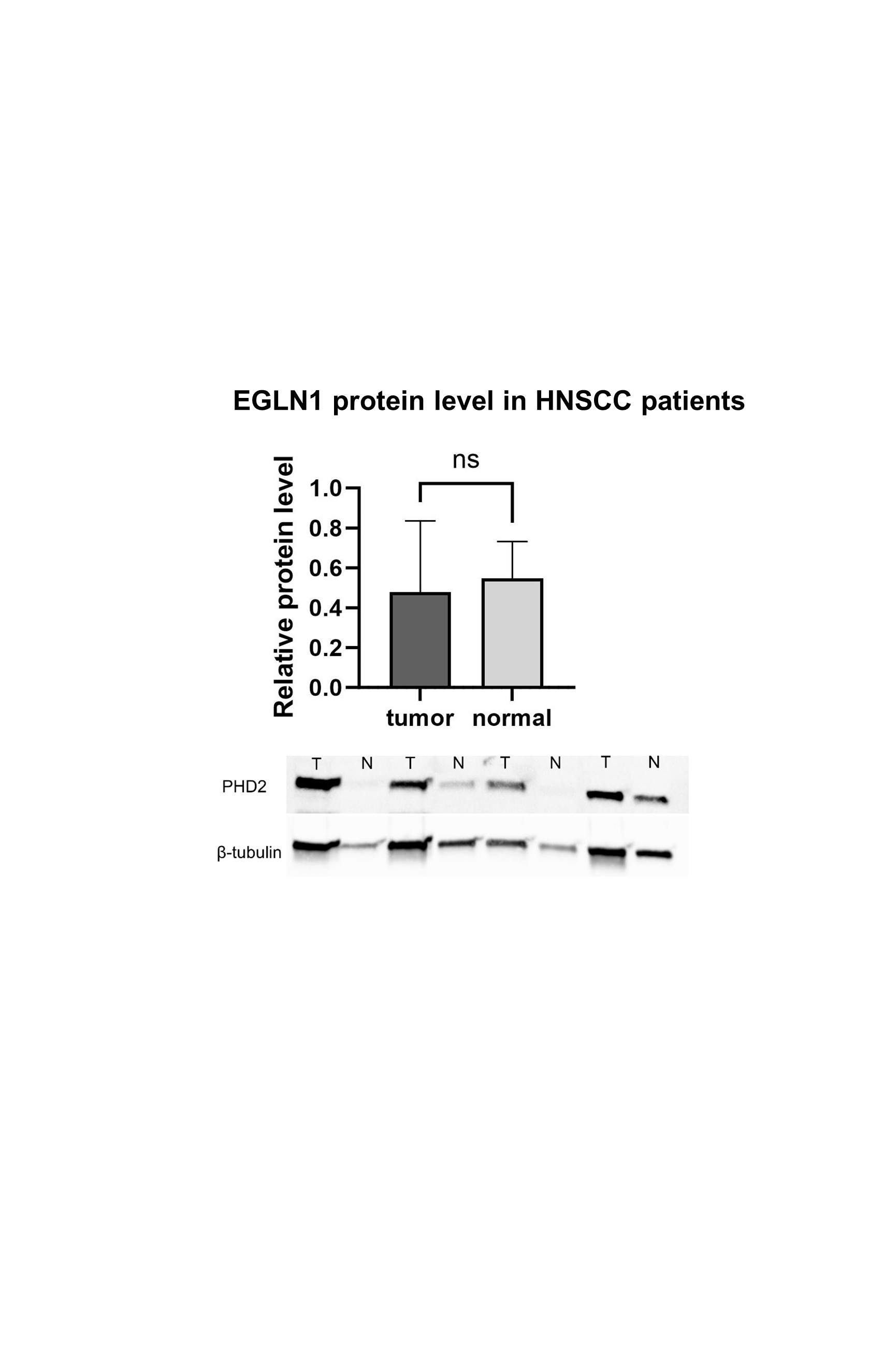

Supplement: Supplementary file 1 [file ijms-25-06495-s001.zip › Supplementary file S2. Protein analysis.jpg]

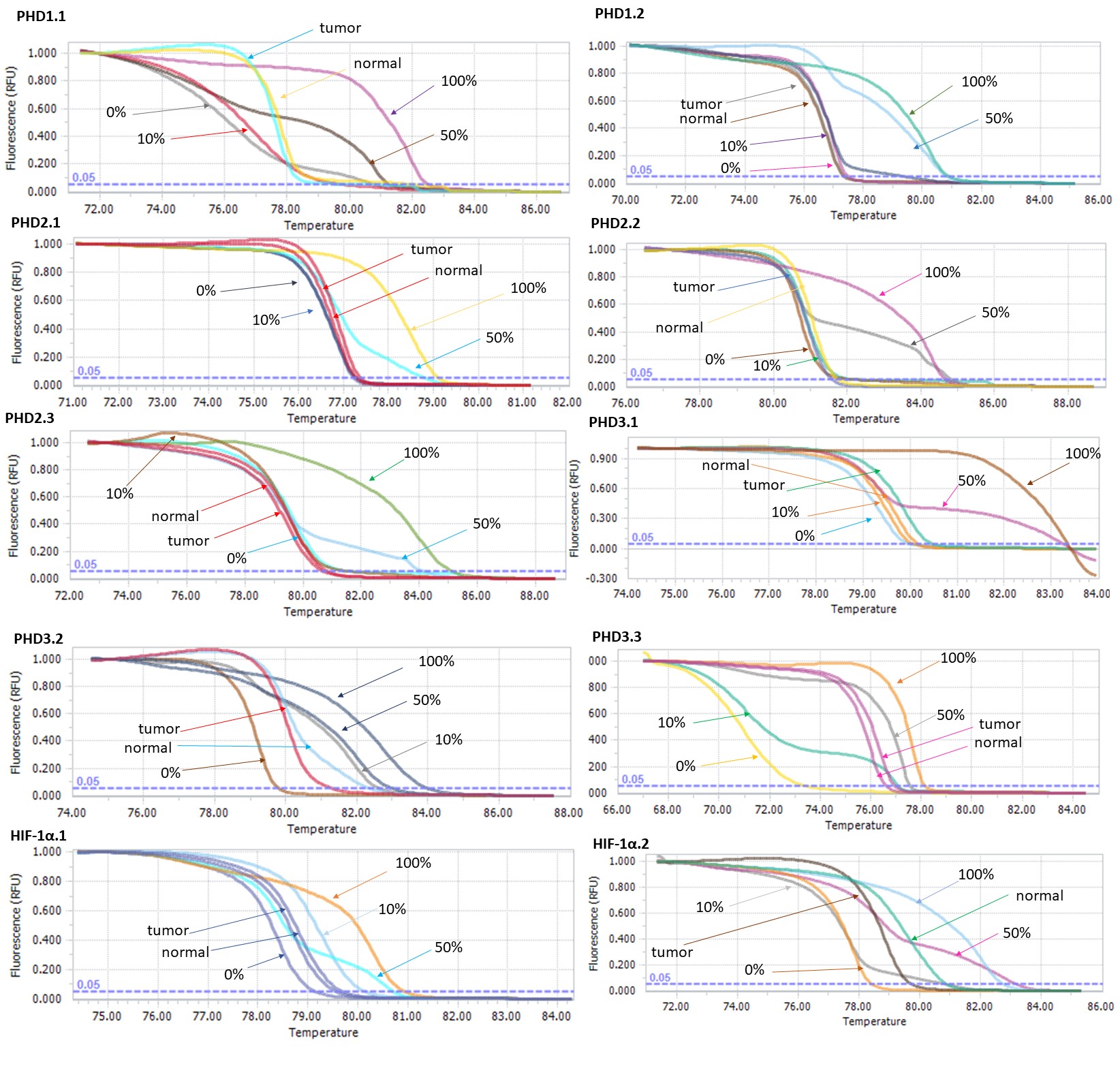

Supplement: Supplementary file 1 [file ijms-25-06495-s001.zip › Supplementary file S5. Representative HRM profiles.jpg]
